# Adrenal Pheochromocytoma: The Great Masquerader

**DOI:** 10.7759/cureus.72483

**Published:** 2024-10-27

**Authors:** Benjith Daniel, Hariharasudhan Sekar, Velmurugan Palaniyandi, Sriram Krishnamoorthy, Josephine Sebastin

**Affiliations:** 1 Urology, Sri Ramachandra Institute of Higher Education and Research, Chennai, IND; 2 Pathology, Sri Ramachandra Institute of Higher Education and Research, Chennai, IND

**Keywords:** adrenal, computed tomography, hypertension, metanephrine, pheochromocytoma

## Abstract

Pheochromocytoma is an uncommon tumor that is often disregarded because of its diverse clinical presentation, which can range from completely unusual symptoms to potentially fatal consequences. Timely diagnosis of pheochromocytoma is crucial due to its treatable form of secondary hypertension and its frequent association with malignancy and metastatic disease. To develop clinical suspicion of pheochromocytoma and establish a rapid diagnosis, physicians must be knowledgeable about pertinent clinical symptoms and diagnostic procedures. The consequences of pheochromocytoma misdiagnosis could be disastrous, even lethal. The purpose of this study is to provide a case series, with a focus on the unique characteristics of the patients, their clinical presentation, the diagnostic assessment, and the intraoperative and postoperative results. A brief overview of pertinent literature and current recommendations is provided to highlight pheochromocytoma screening and suitable diagnostic techniques.

## Introduction

Pheochromocytomas are rare neuroendocrine tumors that originate from chromaffin cells and secrete catecholamines. Nearly 80% of pheochromocytomas are located in the adrenal medulla (mostly benign) and the remaining 20% are in the sympathetic nervous system ganglia, termed paragangliomas or extra-adrenal pheochromocytomas [1]. Pheochromocytoma has an annual incidence of one case for every million population. They are more common in the fourth and fifth decades of life [2]. Familial syndromes associated with pheochromocytoma are Multiple Endocrine Neoplasia (MEN Syndrome - 2A & 2B), Von Hippel Lindau (VHL) syndrome, and Type 1 neurofibromatosis. Nearly 30% of patients are asymptomatic at presentation [3]. The classic clinical triad occurs only in 25% of patients and includes episodic headache, palpitations, and sweating. Paroxysmal hypertension is the most common clinical sign. However, it accounts for less than one percent of hypertensive patients in the general population [4]. Patients who are not treated run the risk of developing cardiovascular morbidity, including heart failure, stroke, catecholamine-induced malignant hypertension, and deadly arrhythmias [5]. For this reason, early detection is crucial. In this case series, we present three cases of adrenal pheochromocytoma with varied clinical presentation.

## Case presentation

Case 1

Presentation: A 34-year-old female, having complaints of giddiness and headache for three months and right lumbar pain for one month initially presented to an outside hospital and was managed conservatively. Because of persistent symptoms despite medical management, she presented to our outpatient department. She had a blood pressure of 170/100 mm Hg and a heart rate of 102 beats per minute on presentation.

**Figure 1:**
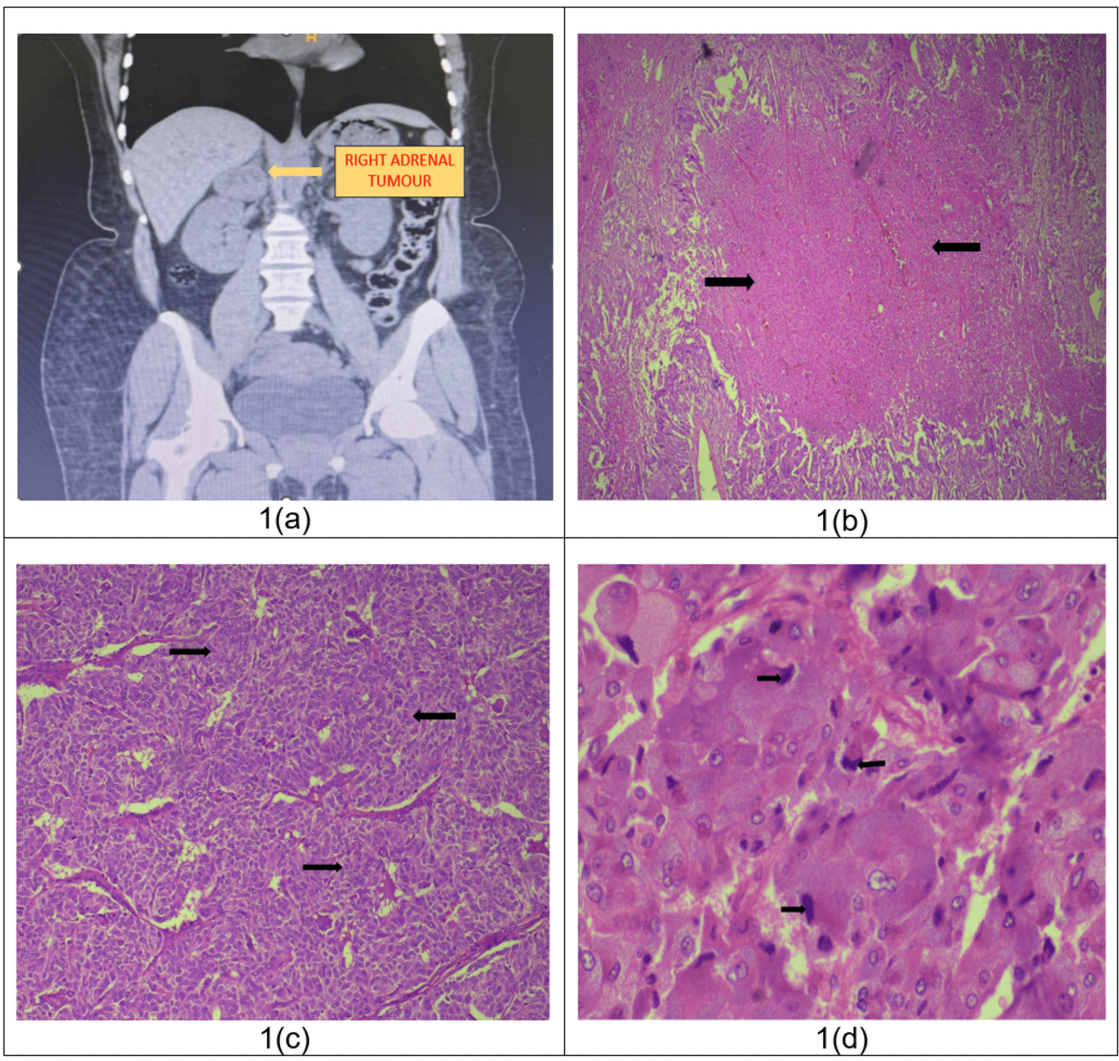
Non-contrast CT image and microphotographs of histopathological sections 1a - Non-contrast computed tomography scan of the abdomen and pelvis (coronal section) showing a well-defined hetero-dense soft tissue density lesion measuring 3.7 x 4.2 x 3.4 cm in the epicenter of the right adrenal gland. 1b - Hematoxylin and Eosin (H & E) stain showing patchy necrosis of tumor cells at 40x magnification* (black arrows)*, 1c - 100x image with H & E stain showing a highly cellular lesion composed of tumor cells arranged in a solid pattern *(black arrows)*, 1d - H & E stain showing mitotic figures at 400x magnification *(black arrows)*.

Investigations: A non-contrast computed tomography scan of the abdomen and pelvis showed a well-defined hetero-dense soft tissue density lesion measuring 3.7 x 4.2 x 3.4 cm in the epicenter of the right adrenal gland (Figure 1a). A functional workup was done which showed elevation of all the markers including plasma metanephrine, 24-hour urine metanephrine, dehydroepiandrosterone sulfate (DHEAS), 24-hour urine vanillylmandelic acid (VMA), and 8 am serum cortisol.

Treatment: Three antihypertensive medications (an α-blocker, a calcium channel blocker, and an angiotensin-converting enzyme inhibitor) were needed to adequately control blood pressure. β-blocker was started after adequate α blockade to control heart rate. The patient underwent a right laparoscopic adrenalectomy and the specimen was sent for histopathological examination. Post-procedure in view of hypotension and need for inotropic support she was shifted to the ICU and then shifted back to the ward once her BP was under control. The patient recovered well and was discharged on postoperative day (POD)-5. Histopathology revealed right adrenal pheochromocytoma with a Pheochromocytoma of the Adrenal gland Scale Score (PASS) of 6 (Figure 1b-1d).

Case 2

Presentation: A 53-year-old male, known diabetic and hypertensive for eight years on regular treatment, having complaints of recurrent hypoglycemic episodes (two to three) in one month and dull aching right lumbar pain for ten days presented to our outpatient department. He had a blood pressure of 140/80 mm Hg, a heart rate of 112 beats per minute, and capillary blood glucose (CBG) upon arrival was 118 mg/dl.

**Figure 2:**
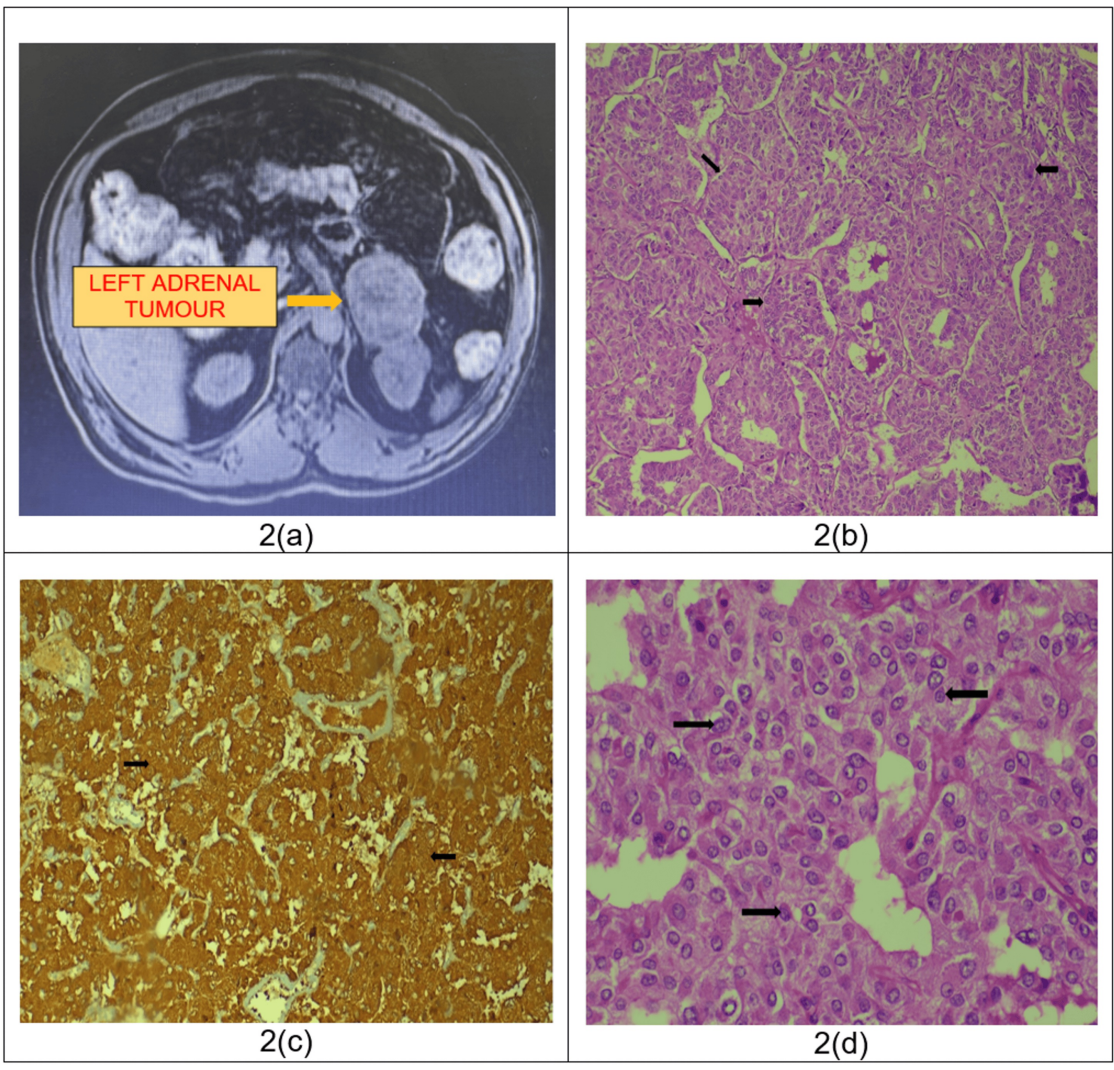
MRI image and microphotographs of histopathological sections 2a - MRI scan of the abdomen and pelvis (axial section) showing a well-defined T2 hetero-dense exophytic lesion measuring 5.8 x 5.5 x 4.6 cm arising from the lateral limb of the left adrenal gland (no visible light bulb sign), 2b - H & E stain showing zellballen pattern of cellular arrangement at 100x magnification *(black arrows)*, 2c - Immunohistochemistry for chromogranin shows cytoplasmic positivity (yellowish brown) at 100x magnification *(black arrows)*, 2d - H & E stain showing monotonous population of cells arranged in sheets at 400x magnification *(black arrows)*.

Investigations: Ultrasound screening showed a suspicious lesion near the upper pole of the left kidney and separate from it. Magnetic resonance imaging (MRI) scan of the abdomen and pelvis showed a well-defined T2 hetero-dense exophytic lesion measuring 5.8 x 5.5 x 4.6 cm arising from the lateral limb of the left adrenal gland without a light bulb appearance (Figure 2a). A pheochromocytoma profile was done which revealed mild elevation of 24-hour urine metanephrine and dehydroepiandrosterone sulfate (DHEAS) with other functional markers within normal limits. 

Treatment: After adequate preoperative measures, the patient underwent left-open adrenalectomy and the specimen was sent for histopathological examination. Post-procedure in view of persistently high BP the patient was shifted to ICU and then shifted back to the ward once his BP was under control. The patient recovered well and was discharged on POD-5. Histopathology revealed left adrenal pheochromocytoma with a PASS score of 3 (Figure 2b-2d).

Case 3

Presentation: A 47-year-old male, known diabetic and hypertensive for three years on regular treatment, presented with complaints of intermittent, dull aching right lumbar pain for 30 days. He was moderately built and nourished and his vitals were stable upon arrival.

**Figure 3:**
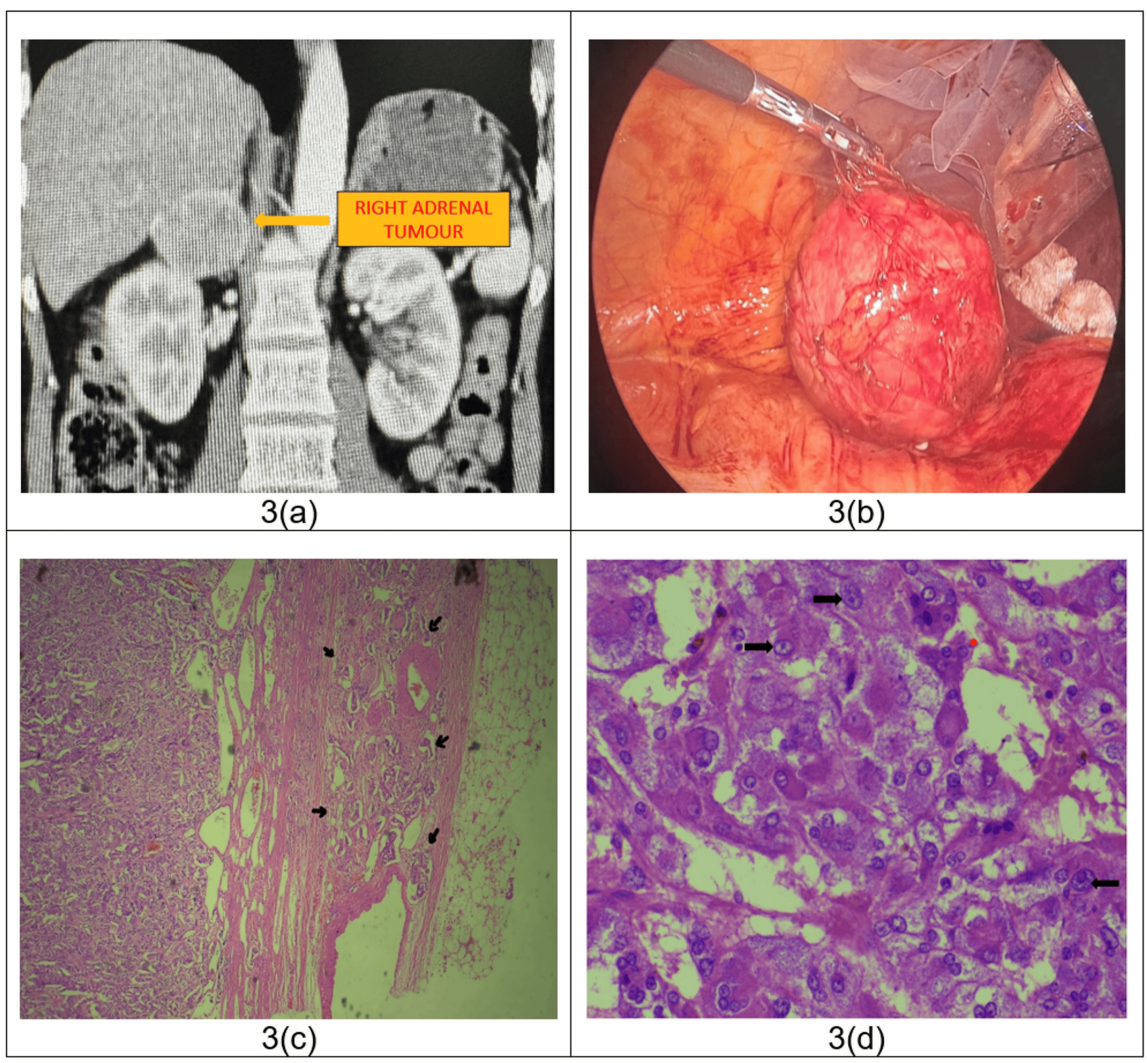
CECT image, intraoperative picture and microphotographs of histopathological sections 3a - Contrast-enhanced computed tomography (CECT) scan of the abdomen and pelvis (coronal section) revealed a well-circumscribed round soft tissue density lesion measuring 5.0 x 4.0 x 5.3 cm entirely replacing the lateral limb of the right adrenal gland, 3b - Intraoperative picture showing right adrenal gland tumor, 3c - H & E stain showing capsular invasion by tumor cells at 40x magnification (*black arrows)*, 3d - H & E stain showing tumor cells with pleomorphic, vesicular nuclei and prominent nucleoli at 400x magnification *(black arrows)*.

Investigations: Ultrasound screening revealed a suspicious lesion located near the upper pole of the right kidney. Contrast-enhanced computed tomography (CECT) scan of the abdomen and pelvis showed a well-circumscribed lesion measuring 5.0 x 4.0 x 5.3 cm entirely replacing the lateral limb of the right adrenal gland. On contrast administration, mildly heterogeneous enhancement was noted with an absolute washout of 70% in the delayed phase. Findings were suggestive of lipid-poor adrenal adenoma (Figure 3a). To exclude pheochromocytoma, we additionally performed a functional workup, which was normal.

Treatment: After pre-anesthetic workup, the patient was taken up for right laparoscopic adrenalectomy. Intraoperatively, as soon as the adrenal was manipulated, the patient’s BP shot up to 220/140 mm Hg and nitroglycerin infusion was started for BP control. We continued dissection and there were no fluctuations in BP. Once the adrenalectomy was completed and we were planning for specimen retrieval [Figure 3(b)] there was sudden profound hypotension (BP - 50/30 mm Hg), for which inotropic supports had to be started. The patient was shifted to ICU post-procedure in view of hypotension and the need for inotropic supports. On POD-1, his BP was under control, inotropic supports were stopped, and shifted back to the ward. The patient was discharged on POD-5. Histopathology revealed right adrenal pheochromocytoma with a PASS score of 4 (Figure 3c-3d).

Table 1 shows the patient demographics, laterality and mode of presentation, preoperative and postoperative blood pressure, number of additional antihypertensives needed to control blood pressure before surgery, and preoperative functional marker values. Table 2 summarizes the case-specific features, including the uniqueness of each case and the lessons learned.

**Table 1:** Demographic data, clinical characteristics and biochemical variables DHEAS - Dehydroepiandrosterone sulfate, VMA - Vanillylmandelic acid, DM - Diabetes mellitus, HTN - hypertension Figures in bold represent the abnormal functional marker values

	Case 1	Case 2	Case 3
Age, years	34	53	47
Gender	Female	Male	Male
Comorbidities	None	DM/HTN	DM/HTN
Laterality	Right	Left	Right
Mode of presentation	Giddiness, Headache, Right Lumbar Pain	Left Lumbar Pain, Recurrent Hypoglycemia	Right Lumbar Pain
Pre-op BP, mm Hg	170/100	140/80	130/80
Post-op BP, mm Hg	110/80	120/70	120/60
No. of additional antihypertensives required (Pre-op)	3	1	-
Functional markers			
Plasma metanephrine, nmol/L	1.8	0.4	0.2
24-hour Urine metanephrine, nmol/day	2380	1580	440
DHEAS, µmol/day	14.8	9.7	3.4
24-hour Urine VMA, µmol/day	68.6	38.2	30.6
Serum Cortisol - 8 AM, nmol/L	862	432	220

**Table 2:** Case-specific data DHEAS - Dehydroepiandrosterone sulfate, VMA - Vanillylmandelic acid, PASS Score - Pheochromocytoma of the Adrenal gland Scaled Score

	Case 1	Case 2	Case 3
Functional markers			
Plasma metanephrine (< 0.5 nmol/L)	Elevated	Normal	Normal
24-hour Urine metanephrine (325 - 1530 nmol/day)	Elevated	Mild elevation	Normal
DHEAS (1.2 – 9.4 µmol/L)	Elevated	Mild elevation	Normal
24-hour Urine VMA (*<* 40.4 µmol/day)	Elevated	Normal	Normal
Serum Cortisol - 8 AM (138-635 nmol/L)	Elevated	Normal	Normal
Intra-op events (if any)	None	High BP that persisted even after removal of the specimen	BP shot up abruptly upon gland manipulation
PASS score	6	3	4
Uniqueness	Classical malignant pheochromocytoma	Benign pheochromocytoma with equivocal presentation	Malignant pheochromocytoma mimicking adenoma
Learning Points	With a high index of suspicion and adequate preoperative preparation, intraoperative catastrophe can be minimized.	Benign pheochromocytoma can sometimes present with equivocal biochemical parameters and without a light bulb appearance on MRI, not convincing enough to be called pheochromocytoma, eventually turning out to be pheochromocytoma.	Normal biochemical parameters with imaging suggestive of adenoma does not rule out pheochromocytoma.

## Discussion

Pheochromocytoma (PC) is referred to as the "great masquerader" due to its ability to generate a wide range of symptoms or none at all [6]. Physicians have faced challenges in managing and treating it ever since Frankel originally described it in 1886. Although, up until 1962, 53% of these tumors were thought to have gone undiagnosed before surgery or autopsy, this number dropped with the development of imaging technology [7]. Erroneous PC diagnoses are linked to severe side effects, morbidity, and death. In a comprehensive investigation, Platts et al. showed that 16 out of 62 patients died as a result of anesthesia and surgery performed in the presence of an undetected PC. Less than 0.5% of patients with hypertension have PC, although it has been shown to account for as much as 4% of patients who have an adrenal incidentaloma at presentation [8]. On the other hand, Mannelli et al. in a retrospective ethnic research involving Italian patients, reported that 11.2% of tumors were incidentalomas of whom 62.5% were normotensive [9].

In our series, the first one was a classical case with elevated functional markers and the patient was taken up for the procedure with adequate preoperative measures. The second patient had an equivocal presentation, with normal to mildly elevated tumor markers, normal BP, and only elevated heart rate. The third patient had the most peculiar presentation with normal functional markers and imaging showing features of lipid-poor adenoma. Intraoperatively with the sudden rise in BP on manipulation of the tumor, there was a suspicion of pheochromocytoma which was later confirmed on histopathological examination.

The type and pattern of catecholamines released from the tumor largely determine how PC presents clinically [10]. Pheochromocytoma can be asymptomatic. On the other hand, palpitations, perspiration, and episodic headaches are the traditional trio of PC presentations. Breathlessness, anxiety, chest discomfort, nausea, vomiting, tremors, and paresthesia are additional symptoms associated with PC. It is important to remember that the subclinical picture does not rule out the possibility of a hypertensive crisis. Malignant PC can also exhibit systemic symptoms or clinical manifestations associated with metastatic disease, such as pain in the bones due to metastatic spread, in addition to the symptoms and indicators already listed. The most common sites for metastases are the lungs, liver, and bones [11].

Biochemistry tests continue to be essential for diagnosis. Measures of free metanephrines (metanephrine and normetanephrine) in plasma and urinary fractionated metanephrines are first used to identify pheochromocytomas, which are confirmed by specific imaging techniques [12]. Due to the low prevalence of PC, false positive biochemical test results are frequent and pose unique challenges. Testing ought to be conducted again to confirm the findings. Imaging studies such as CT and MRI are useful as radiologic approaches once a biochemical diagnosis has been made. Most pheochromocytomas larger than 5 mm may be localized with sufficient sensitivity using CT and MRI. CT and MRI scans show 90%-100% sensitivity and 70%-80% specificity. Due to the moderate specificity rates of MRI and CT imaging, confirmatory studies using iodine-123-labeled metaiodobenzylguanidine (123MIBG) or 131MIBG are advised [13]. However, Adler et al. recommended that scintigraphic studies were not necessary if there was no suggestion of familial or bilateral disease [14].

Positron emission tomography (PET) imaging with the 18F-fluorodeoxyglucose positron emission tomography (18F-FDG PET) is often reserved for the identification of extra-adrenal paragangliomas or large tumors to rule out metastases [15]. Undiagnosed imaging tests and a silent hormone profile were the main contributing factors to the misdiagnosis of pheochromocytoma. Massive catecholamine release may happen during PC surgical manipulation, which could lead to multiple organ failure, cerebral vascular accident, myocardial infarction, pulmonary edema, and hypertensive crisis [16]. To lower the perioperative morbidity and mortality in these patients, preoperative planning is crucial. The Endocrine Society advises patients undergoing pheochromocytoma excision to take alpha-blockade, drink plenty of water, and consume salt to reduce hemodynamic instability during tumor manipulation [17]. Once alpha-adrenergic blockade has successfully brought blood pressure back to normal, beta-antagonists should be given to prevent symptoms such as tachycardia [18]. In 20-70% of cases, hypotension might develop following surgery. Following the removal of the tumor, the abrupt withdrawal of catecholamines also causes rebound hyperinsulinemia, which, combined with the already reduced glycogen stores, can cause severe hypoglycemia during the recovery phase. Therefore, following surgery, blood sugar and arterial pressure monitoring are required [19].

## Conclusions

A high index of suspicion and thorough evaluation is very important in all suspected cases of pheochromocytoma. To develop clinical suspicion of pheochromocytoma and establish a rapid diagnosis, physicians must be knowledgeable about pertinent clinical symptoms and diagnostic procedures. Timely diagnosis of pheochromocytoma is crucial due to its treatable form of secondary hypertension and its frequent association with malignancy and metastatic disease. Pheochromocytoma in normotensive patients with normal biochemical markers is not uncommon. Even if nuclear imaging is not done in patients with classical presentation of pheochromocytoma, it should be strongly considered in patients with equivocal presentation to prevent deleterious on-table complications. The consequences of pheochromocytoma misdiagnosis could be disastrous, even lethal.
